# Chemical Structures and Bioactivities of Sulfated Polysaccharides from Marine Algae

**DOI:** 10.3390/md9020196

**Published:** 2011-02-08

**Authors:** Guangling Jiao, Guangli Yu, Junzeng Zhang, H. Stephen Ewart

**Affiliations:** 1 National Research Council Canada, Institute for Marine Biosciences, Halifax, NS, B3H 3Z1, Canada; Email: guangling.jiao@nrc-cnrc.gc.ca; 2 Key Laboratory of Marine Drugs, Ministry of Education, School of Medicine and Pharmacy, Ocean University of China, Qingdao, Shandong 266003, China; 3 National Research Council Canada, Institute for Nutrisciences and Health, Charlottetown, PEI, C1A 4P3, Canada; Email: junzeng.zhang@nrc-cnrc.gc.ca

**Keywords:** sulfated polysaccharide, marine macroalgae, carbohydrate structure, bioactivity, oligosaccharides

## Abstract

Sulfated polysaccharides and their lower molecular weight oligosaccharide derivatives from marine macroalgae have been shown to possess a variety of biological activities. The present paper will review the recent progress in research on the structural chemistry and the bioactivities of these marine algal biomaterials. In particular, it will provide an update on the structural chemistry of the major sulfated polysaccharides synthesized by seaweeds including the galactans (e.g., agarans and carrageenans), ulvans, and fucans. It will then review the recent findings on the anticoagulant/antithrombotic, antiviral, immuno-inflammatory, antilipidemic and antioxidant activities of sulfated polysaccharides and their potential for therapeutic application.

## 1. Introduction

Many species of seaweed (marine macroalgae) are used as food and they have also found use in traditional medicine because of their perceived health benefits. Seaweeds are rich sources of sulfated polysaccharides, including some that have become valuable additives in the food industry because of their rheological properties as gelling and thickening agents (e.g., carrageenan). In addition, sulfated polysaccharides are recognized to possess a number of biological activities including anticoagulant, antiviral, and immuno-inflammatory activities that might find relevance in nutraceutical/functional food, cosmetic/cosmeceutical and pharmaceutical applications. 

In this review, we will examine current progress in research on sulfated polysaccharides, relating to their structural diversity, bioactivities and mechanisms of action. The literature contains vast information on the structure and bioactivities of marine sulfated polysaccharides that cannot be completely considered in this short review. There are several excellent reviews where the reader may find additional information on various aspects of this subject [1,2,3,4,5,6,7,8,9]. 

## 2. Structural Diversity of Algal Sulfated Polysaccharides

### 2.1. Carrageenans and Agarans from Red Algae

Red seaweed galactans are of great commercial importance as they are used widely in the food industry because of their rheological properties as gelling and thickening agents. These sulfated polysaccharides are primarily classified as agarans and carrageenans based on their stereochemistry, specifically galactans with 4-linked α-galactose residues of the L-series are termed agarans and those of the D-series are termed carrageneens [10]. Thus, carrageenans are high molecular weight sulfated D-galactans composed of repeating disaccharide units with alternating 3-linked β-D-galactopyranose (G-units) and 4-linked α-galactopyranose (D-units) or 3,6-anhydro-α-galactopyranose (AnGal-units). 

**Figure 1:**
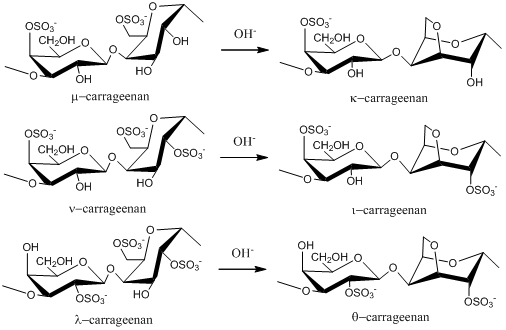
Repeating disaccharide units of different types of carrageenan and their transformation by treatment with alkali.

Carrageenans are normally classified according to their structural characteristics, including their sulfation patterns and the presence or absence of AnGal on D-units. There are at least 15 different carrageenan structures [2]. The most industrially relevant carrageenans are κ, ι and λ forms, the structures of which are illustrated in Figure 1. The major source of κ-carrageenan is the red seaweed *Kappaphycus alvarezii*  [11]. Its structure was reported as alternating 3-linked β-D-galactose 4-sulfate and 4-linked AnGal units [12]. The ι-carrageenans have an additional sulfate group on C2(O) of the AnGal residue, resulting in two sulfates per disaccharide repeating unit. Funami *et al.* examined ι-carrageenan extracted from *Eucheuma spinosum* using atomic force microscopy and suggested that ι-carrageenans were more homogeneous and flexible than κ-carrageenans [13]. The λ-carrageenans have three sulfate groups per disaccharide unit with the third sulfate group of this form at the C6 position of the 4-linked residue, but there is no 3,6-anhydride bridge on the 4-linked residues. Lambda-carrageenan is obtained from species of the *Gigartina* and *Chondrus* genera [14]. 

Alternative forms of carrageenan can be obtained by chemical modification. For example, formation of an anhydride bridge in λ-carrageenan can be induced by alkali modification to produce θ-carrageenan (Figure 1). Extraction of λ-carrageenan from hand-sorted tetrasporophytes of *Gigartina skottsbergii* and subsequent treatment of the extract with alkaline borohydride resulted in conversion of the 4-linked residues to the 3,6-anhydride ring form, yielding θ-carrageenan with no detectable contamination of κ- or ι-carrageenans [15]. 

Natural carrageenans typically occur as mixtures of different hybrid types, such as κ/β-hybrids [16], κ/ι-hybrids [17,18,19,20], κ/μ-hybrids [21], or ν/ι-hybrids [22]. Additionally, methyl or pyruvic acid acetal constituents and the presence of small amount of other sugars can add to the structural complexity [23].

One of the best studied agarans is porphyran [24], obtained from *Porphyra* species of red algae including *Porphyra capensis* [25] and *P. haitanensis*  [26,27]. Porphyran typical exhibits a linear backbone of alternating 3-linked β-D-galactose and 4-linked α-L-galactose-6-sulfate or 3,6-anhydro-α-L-galactose units. Sulfated agarans of a similar linear form are synthesized by *Polysiphonia* species, such as *P. strictissima*, *P. abscissoides* [28], *P. nigrescens*  [29],and *P. atterima* [30]. The regular agaran backbone may be interrupted by different *O*-linked substitutions in addition to sulfate including methyl and xylosyl groups adding to the structural diversity. For example, Prado *et al.* recently reported that the sulfated agaran from *P. nigrescens* is highly substituted on the C-6 of β-D-galactose with sulfate, but methyl ether and β-D-xylose residues were also present [29]. Agaran from *Acanthophora spicifera*, is highly sulfated at the C-2 position of β-D-galactose units, with some of the residues being 4,6-pyruvylated [31]. This agaran also contains small amounts of xylose and sulfated xylose residues [31,32]. 

In addition to carrageenans and agarans, there are also red seaweed sulfated polysaccharides that have 4-linked D- and L-galactose sugars distributed within the same polysaccharide molecules, so-called DL-hybrids, and others with various substitutions involving sulfate groups, pyruvic acid ketals, and methoxyl groups [33]. Indeed, native polysaccharides are rarely in their uniform or “ideal” form. For example, we recently reported on the existence of a carrageenan-like sulfated galactan from *Furcellaria lumbricalis* composed of κ/β-carrageenan units, non-sulfated galactan units, and also smaller units containing 3-*O*-methyl-galactose [16]. Another example of non-ideal sulfated galactans are xylogalactans, first described in the red seaweed *Corallina officinalis* and termed corallinan [34]*,* which are agarans that have β-D-xylosyl groups attached at the *O*-6 position of D-galactose units [35,36,37].

It should be noted that red seaweeds also produce other types of sulfated polysaccharides including those with mannose in their backbones [38,39]. For example, Mandal *et al.* described xylomannnan from *Scinaia hatei* consisting primarily of a backbone of α-(1→3)-linked D-mannose residues substituted at C-6, C-4, and C-2 with β-D-xylosyl residues [39].

### 2.2. Sulfated Polysaccharides from Green Algae

**Figure 2:**
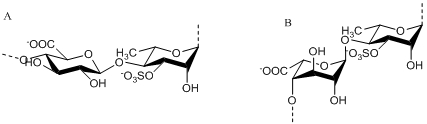
The main repeating disaccharide units of ulvan. A. [→4)-β-D-Glc*p*-(1→4)-α-L-Rha*p*3S-(1→]_n_; B. [→4)-α-L-Ido*p*-(1→4)-α-L-Rha*p*3S-(1→]_n_.

Ulvan is the major water-soluble polysaccharide found in green seaweed of the order Ulvales (*Ulva* and *Enteromorpha* sp.) that has sulfate, rhamnose, xylose, iduronic and glucuronic acids as main constituents [40,41]. As reviewed by Lahaye and Robic, ulvan structure shows great complexity and variability as evidenced by the numerous oligosaccharide repeating structural units identified in native and chemically modified ulvan preparations [3]. The main repeating disaccharide units reported are ulvanobiouronic acid 3-sulfate types containing either glucuronic or iduronic acid (Figure 2). Additionally, minor repeat units have been reported that contain sulfated xylose replacing the uronic acid or glucuronic acid as a branch on *O*-2 of the rhamnose-3-sulfate [40,42]. 

Although the most common source of sulfated galactans is red macroalgae, some green algae, particularly *Codium* species, are a significant source of sulfated galactans [43,44,45]. Sulfated galactans from green algae tend to be more complex and heterogeneous in structure than their counterparts from red algae. For example, *C. fragile* and *C. cylindricum* contain sulfated arabinogalactan and sulfated glucogalactan, respectively [43,46]. Bilan *et al.* reported a highly ramified sulfated galactan from *C. yezoense* that contained a linear backbone of 3-linked β-D-galactopyranose residues containing short oligosaccharides branches through (1→6) linkages [47]. Sulfate groups were found mainly at C-4 and in minor amounts at C-6. Polysaccharides containing sulfated galactans from other green seaweeds including *Caulerpa* and *Ulva* have been reported [48,49], but the galactans are minor components. 

A variety of other forms of sulfated polysaccharides are synthesized by green seaweeds [41,50,51,52,53]. This includes, for example, a water-soluble heteroglycuronan from *Enteromorpha compressa*, composed of (1→2,4)-linked rhamnose, (1→4)-linked xylose, and (1→4)-linked glucuronic acid units [52]. Sulfate groups, when present, were situated at the C-3 of rhamnose and the C-2 of xylose. Recently, a rhamnan sulfate from *Monostroma nitidum* was shown to consist primarily of α-1,3-linked and α-1,2-linked rhamnose residues [51]. 

### 2.3. Fucose-containing Sulfated Polysaccharides from Brown Algae

**Figure 3:**
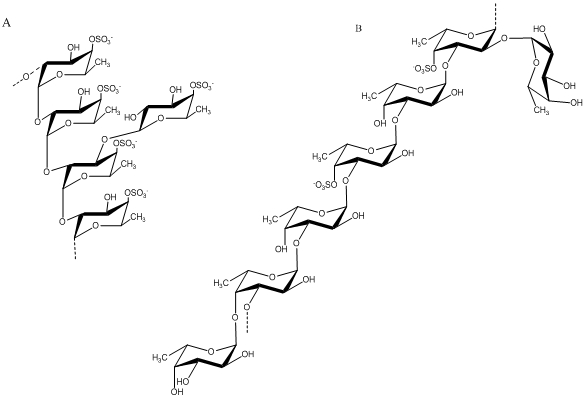
Structure of fucoidan prepared from *F. vesiculosus* by Percival (**A**) [55] and Patankar (**B**) [56].

Fucans are sulfated polysaccharides that are composed of a fucose backbone. One of the best studied fucans from brown algae is fucoidan, which was first isolated by Kylin in 1913 [54]. The fucoidan from *Fucus vesiculosus* has been available commercially for decades (Sigma-Aldrich Chemical Company, St. Louis, MO, U.S.). Early work on its structure showed that it contained primarily (1→2) linked 4-*O*-sulfated fucopyranose residues [55]. However, 3-linked fucose with 4-sulfated groups were subsequently reported to be present on some of the fucose residues [56]. Additionally, it was determined to contain branches every 2-3 fucose residues. These early structures of fucoidan from *F. vesiculosus* are illustrated in Figure 3. Subsequently, Chevolot and colleagues reported that the fucoidan from *F.* *vesiculosus* and *Ascophyllum nodosum* contains a predominant disaccharide motif containing sulfate at the 2-position of the 3-linked fucose and sulfate groups on the 2- and 3-positions of the 4-linked fucose [57].

Fucans can differ in structure among algal species and can vary even within the same species. Because of the heterogeneity in structures within seaweed, differing extraction conditions used by researchers can give rise to the isolation of distinct fucan forms [4]. Fucans have been classified into two groups [58]. One group includes the fucans from *Laminaria saccharina*, *L. digitata*, *Analipus japonicus*, *Cladosiphon okamuranus*, and *Chorda filum* that have their central chains composed of (1→3)-linked α-L-fucopyranose residues. A second group included fucans isolated from *Ascophyllum nodosum* and *Fucus* species that have their central chains composed of repeating (1→3)- and (1→4)-linked α-L-fucopyranose residues. However, many studies have revealed more complex fucans some with branching structures. A fucoidan isolated from *Turbinaria conoides* was shown to be highly complex, with 33-34% terminals, 27-28% linked and 21-22% branched in the (1→3)-linked main chain [59]. Representative fucan structures are illustrated in Table 1.

**Table 1:** Representative structures reported for fucans from brown algae.

**Species**	**Fucoidan structures**	**Reference**
*Analipus japonicus*	3 (4Fuc*p*) and 1 (2Fuc*p*) per ten (1→3)-α-L-Fuc*p*(2/4SO_3_^-^) residues	Bilan* et al.*, 2007 [60]
*Ascophyllum nodosum*	→3)-α-L-Fuc*p*(2SO_3_^-^)-(1→4)-α-L-Fuc*p*(2,3-diSO_3_^-^)-(1→	Chevolot *et al.*, 2001 [57]
*A. nodosum*	(1→3)-α-L-Fuc*p* and a few (1→4)-α-L-Fuc*p* with (1→3)-α-L-(2 and/or 4 Fuc*p*)	Marais *et al.*, 2001 [61]
*Chorda filum*	-[→3)-α-L-Fuc*p*-(1-]_3_→3)-α-L-Fuc*p*(2Fuc*p*)-(1→	Chizhov *et al.*, 1999 [62]
*Fucus distichus L.*	→3)-α-L-Fuc*p*-(2,4-diSO_3_^−^)-(1→4)-α-L-Fuc*p*-(2SO_3_^−^)-(1→	Bilan *et al.*, 2004 [63]
*F. evanescens*	→3)-α-L-Fuc*p*(2SO_3_^-^)-(1→4)-α-L-Fuc*p*(2SO_3_^-^)-(1→	Bilan *et al.*, 2002 [64]
*F. serratus L*	→3)-α-L-Fuc*p*(2R_1_,4R_2_)-(1→4)-α-L-Fuc*p*(2SO_3_^−^)-(1→	Bilan *et al.*, 2006 [65]
	(~50%): R_1_ = SO_3_^−^, R_2_ = H	
	(~50%): R_1_ = H, R_2_ = α-L-Fuc*p*-(1→4)-α-L-Fuc*p*(2SO_3_^−^)-(1→3)-α-L-Fuc*p*(2SO_3_^−^)-(1→	
*Laminaria saccharina*	→3)-α-L-Fuc*p*(4SO_3_^-^)-(1→ and additionally →3)-α-L-Fuc*p*(4SO_3_^- ^or 2Fuc*p*)-(1→	Usov *et al.*, 1998 [66]
*Stoechospermum marginatum*	→3)-α-L-Fuc*p*(2/4SO_3_^−^)-(1→ and	Adhikari *et al.*, 2006 [67]
	→4)-α-L-Fuc*p*(2SO_3_^−^)-(1→	

Many fucans from brown algae contain small amounts of other monosaccharides, including glucose [68], galactose [69], mannose [70,71,72], xylose [69,73], uronic acids [68,74] and also acetyl groups [75]. 

## 3. Approaches in Structural Analysis of Algal Sulfated Polysaccharides

### 3.1. Desulfation and Methylation for Structure Analysis

Structure analysis of sulfated polysaccharides requires determination of the attached sulfate esters along their backbones and the glycosidic linkage types. Methylation is used to determine linkages between monsaccharides. By comparing the methylation of native polysaccharides to that of their desulfated counterparts, the positions of the sulfate groups can be determined. Thus, hydrolysis of the permethylated, desulfated polysaccharides, yields partially methylated monosaccharides, which following acetylation are separated and identified on gas chromatography/mass spectra [76]. 

Accurate structural determination requires desulfation of the polysaccharide without cleavage of the polysaccharides chain linkages. Often a solvolytic desulfation procedure is used wherein the polysaccharide as a pyridinium salt is heated in dimethyl sulfoxide [47,77,78]. Desulfation of fucoidan by methyl sulfoxide-pyridine is rapid and complete, resulting in higher yields with little degradation. However, a method using chlorotrimethylsilane (CTMS) for treatment of pyridinium salts is more appropriate for desulfation of sulfated galactans of both the agaran and carrageenan families [79]. Other approaches for desulfation that have been used involve methanolic hydrogen chloride [80], silylating reagents [81], and pyromellitic acid [82]. Chemical desulfation is relatively non-specific and usually results in a significant loss of sample material. The use of sulfatases represents a more specific approach to desulfation and would be advantageous in structural studies, yet such enzymatic approaches do not appear to be a frequent method of choice [7,83]. The reason for this is unclear but could be due to the lack of commercially available enzymes of the required specificity.

The typical methylation procedure is straightforward, involving treating the desulfated polysaccharide sample with methyl iodide in the presence of solid base, usually sodium hydroxide, in methyl sulfoxide and the procedure can be repeated to obtain a complete methylation [51,62,77,84,85].

### 3.2. Structural Analysis by Using NMR and MS

A powerful tool for the structural elucidation of sulfated polysaccharides is NMR spectroscopy, which can provide structural details such as the monosaccharide components, linkages, anomeric configurations, and positions of branching or sulfations. This can be done by combining various 1D and 2D-NMR techniques. The use of NMR in the structural analysis of red algal galactans and green algal ulvans has been considerable [3,86]. In part, this has been due to the relative high proportion of repeating sequences (identical sulfation pattern) in these polysaccharides that make them amenable to analysis by ^13^C-NMR. For example, Gonçalves and colleagues described the structural elucidation by NMR of positional isomers of sulfated oligosaccharides obtained from agarans and carrageenans [87]. This entailed partial reductive hydrolysis to produce oligosaccharides from repetitive galactans followed by separation by anion exchange and gel-filtration chromatography prior to 1D and 2D NMR analysis.

Effort has also been made to elucidate the structures of fucans by NMR. Usov *et al.*, by 1D-NMR, found the sulfated fucans from *Saccharina latissima* (formerly *Laminaria saccharina*) to be 1→3 linked α-L-fucopyranose with a sulfate group at C4 and branched at C2 [66]. More recently, they confirmed the complex structure of this fucoidan with a more detailed structural investigation by 2D-NMR [88]. These studies also revealed the presence of three additional sulfated polysaccharide types, a fucogalactan, a fucoglucuronomannan and a fucoglucuronan, that appear to occur in minor amounts in their preparation. 

Mass spectrometry (MS) is valuable in the structural analysis of polysaccharides, as it generates accurate molecular mass data for oligosaccharides and it can also provide sequence information. Compared with other analytical techniques, mass spectrometric methods have several advantages, including low sample consumption (e.g., picomole quantities) and short analysis time. While analysis of sulfated polysaccharides by MS can be problematic due to the labile nature of the sulfate groups, approaches based on electrospray ionization and matrix-assisted laser desorption/ionization (MALDI) are increasingly being developed to characterize sulfated oligosaccharides [89,90,91,92]. Negative-ion ESI-CID-MS/MS was used to characterize oligosaccharide fragments derived from mild hydrolysis of κ-carrageenan that revealed highly ordered disaccharide repeats leading to a complete series of exclusively odd-numbered oligosaccharides [92]. Similarly, fucan oligosaccharides from *A. nodosum*, including a highly sulfated pentasaccharide, were analyzed successfully by negative ion ESI-MS [89].

### 3.3. Oversulfation of Algal Polysaccharides

Structure modification of sulfated polysaccharides, such as desulfation, oversulfation, acetylation and benzoylation, would allow the development of new and possibly more effective derivatives of naturally occurring polysaccharides [93,94,95]. For example, benzoylated derivatives of native ulvan from *Ulva pertusa* exhibited enhanced antioxidant properties [94]. Certainly, the most frequent structural modification to sulfate polysaccharides is oversulfation due to the typically strong positive correlation between their sulfate content and biological activity (discussed further in Section 4). A number of methods have been developed for polysaccharide oversulfation, such as treatment with sulfuric acid, sulfur trioxide-pyridine, chlorosulfonic acid-pyridine, dimethylformamide and sulfur trioxide-dimethylamine [96,97,98,99,100,101,102].

### 3.4. Molecular Size Modification of Algal Sulfated Polysaccharides

Lower molecular weight algal sulfated polysaccharides can be prepared by chemical, physical or enzymatic means to obtain oligosaccharides with more diverse bioactivities. For example, linear or branched sulfated galactans and fucans can be cleaved by mild acid hydrolysis [99,102] or by a radical process involving a hydrogen peroxide-cupric redox system [98,101]. The chemical methods are easy to perform but lack specificity. Minor changes in temperature or acidity can lead to variations in oligosaccharide sizes and sulfation patterns, while strong acid may alter the sulfation pattern or destroy the polysaccharide chain [97]. Enzymatic degradation of sulfated polysaccharides can be achieved by selecting enzymes such as hydrolases, fucoidanases (EC 3.2.1.44), α-L-fucosidases (EC 3.2.1.51) and galactosidases [96,103] to target glycosidic bonds while preserving the sulfate groups [100]. Fucoidanases and galactosidases have been identified in marine invertebrates and microorganisms [83,104,105,106,107,108]. Some bioactivities of lower molecular weight oligosaccharides prepared from algal sulfated polysaccharides are highlighted in Table 2.

**Table 2:** Biological activities of selected low molecular weight oligosaccharides derived from algal sulfated polysaccharides.

**Modification methods**	**Source**	**Applications**	**Reference**
Chemical	*Ascophyllum nodosum*	Antithrombotic activity	Colliec-Jouault *et al.*, 2003 [109]
	*Botryocladia occidentalis*	Anti-venom activity	Toyama *et al.*, 2010 [110]
	*Furcellaria lumbricalis*	Immunostimulation activity	Yang *et al.*, 2011 [16]
	*Solieria chordalis*	Immunostimulation activity	Bondu *et al.*, 2010 [111]
Enzymatic	*Chondrus ocellatus*	Anti-tumor activity	Mou *et al.*, 2003 [112]
	*Nemacystus decipieus*	Anticoagulant activity	Kitamura *et al.*, 1992 [113]
	*Pelvetia canaliculata*	Antiviral activity	Klarzynski *et al.*, 2003 [114]
	*Undaria pinnatifida*	Anticoagulant activity	Kim *et al.*, 2010 [115]

## 4. Bioactivity and Structure-Activity Relationship

Due to the difficulties in identifying the chemical structure of algal sulfated polysaccharides, the relation between their structures and biological activities is not completely understood. One of the research approaches in establishing structure-function relationships has been to make inferences based on information obtained from studies of invertebrate sulfated polysaccharides that have a regular structure and so are more easily studied. For example, Pereira and colleagues compared branched fucans from brown seaweeds with the more simple linear versions obtained from sea cucumber and sea urchins [116]. The anticoagulant activity of seaweed fucans was shown to depend on their molecular weight, the extent of sulfation, and the distribution of sulfate groups in the repeating units. Interestingly, the algal fucans were found to inhibit coagulation through a direct inhibition of thrombin whereas the invertebrate fucans inhibited the enzyme indirectly with a requirement for antithrombin and heparin cofactor II.

In this section, we will highlight the key biological activities that have been reported for algal sulfated polysaccharides, the current knowledge regarding their mode of action and the structural requirements necessary to elicit these effects.

### 4.1. Anticoagulant and Antithrombotic Activities

Probably the most widely recognized and studied bioactivity of marine sulfated polysaccharides is the heparin-like anticoagulant activity exhibited by fucoidans and other fucans of brown seaweeds. This was first reported for fucoidan isolated from *F. vesiculosus* by Springer and colleagues who found inhibition of fibrin clot formation and antithrombin activity [117,118]. Since then, studies on fucans from various seaweeds have revealed anticoagulant and antithrombotic activities and are discussed in several recent reviews [1,4,6].

The basis for these activities is not completely understood, but a number of investigations suggest more than one mechanism of action including direct and indirect inhibition of thrombin through the activation of thrombin inhibitors (e.g antithrombin and heparin cofactor II) [116,119,120,121]. Recently, Cumashi and colleagues reported that fucans from 10 brown seaweeds each prolonged the clotting time of human plasma; however, only five of these fucans had significant activity against thrombin-induced platelet aggregation [122]. While the results of the latter assay are suggestive of a direct action of certain fucans on thrombin, the authors pointed out that an interfering action of thrombin binding to its receptors on platelets could not be ruled out. 

The general structural features of fucans that are important in their anticoagulation activity include sugar composition, molecular weight, sulfation level and the position of sulfate groups on the sugar backbone [70,123,124,125]. For example, Nishino and colleagues found that a higher content of fucose and sulfate groups coincided with higher anticoagulant activities in sulfated polysaccharide fractions from *E. kurome* [70]. They also showed that anticoagulation activity of fucans was positively correlated with sulfate content and that only fucans with a sulfate-total sugar residue ratio greater than one possessed significant activity [93,123]. Higher molecular weight fucans (e.g., 27 and 58 kDa) showed greater anticoagulant activity than lower molecular weight ones (~10 kDa) [126]. The relationship between molecular weight of sulfated polysaccharides and their anticoagulant activity was also considered by Pomin and colleagues who reported that linear, sulfated fucan required significantly longer chains than mammalian glycosaminoglycans to achieve anticoagulant activity [125]. Selective cleavage to reduce molecular size of the fucan by only a modest amount dramatically reduced its effect on thrombin inactivation mediated by heparin cofactor II. Lower molecular weight fucans appear to bind to heparin cofactor II but, unlike the native (full length) fucan, were unable to effectively facilitate the heparin cofactor II interaction with thrombin [125]. Chevolot *et al.* reported on the importance of sulfate group location on the sugar residues for anticoagulant activity [57,62]. Studying the fucoidan from *A. nodosum*, they found that anticoagulant activity required 2-*O*-sulfated and 2,3-*O* disulfated fucose residues, whereas sulfation at the *O*-4 position did not appear necessary. 

Marine sulfated polysaccharides other than fucans have also been shown to possess anticoagulant activities. Reports include sulfated galactan and ulvan-like sulfated polysaccharides obtained from green algae, in particular from species of *Codium* and *Ulva* [44,45,49,127,128]. For example, Mao *et al.* described a sulfated polysaccharide from *U. conglobata* with high rhamnose content and 35% sulfate ester that prolonged clotting time through what appeared to be direct inhibition of thrombin and modulation of heparin cofactor II [49]. Hayakawa *et al.* tested sulfated polysaccharides from 23 green algae species for anticoagulant activity and discovered a high rhamnose-containing sulfated polysaccharide from *Monostroma nitidum*, the purified version of which was more potent than standard heparin [127]. 

Red seaweeds have also yielded a number of sulfated polysaccharides with potent anticoagulant activities [129,130,131]. Studies on a sulfated galactan from the red seaweed *Botryocladia occidentalis* are particularly illustrative. Farias *et al.* reported that a 2,3-di-*O*-sulfated D-galactan from *B. occidentalis* exhibited anticoagulant activity, comparable to heparin, which appeared to be due to inhibition of thrombin and factor X. Its activity was more potent than similar sulfated galactans, from invertebrate sources, that had only one sulfate per galactose residue [129]. A similar polysaccharide chain from *G. crinale*, but with lower amounts of 2, 3-di-*O*-sulfated D-galactose, was less potent in a clotting time assay when compared with that from *B. occidentalis* [131]. The two sulfated polysaccharides did not differ in thrombin inhibition mediated by antithrombin; however, in assays where heparin cofactor II was used in place of antithrombin, the sulfated galactan from *G. crinale* was less inhibitory than that from *B.* *occidentalis.* Yet the sulfated galactan from *G. crinale* was a more potent anticoagulant than that from *B. occidentalis* when Factor X was the target protease. These observations suggested that the proportion and/or the distribution of 2,3-di-sulfated galactose along the polysaccharide chain modulate the interaction of the polysaccharides with specific proteases in the coagulation system. Recently, Glauser *et al.* showed that the 2,3-disulfated galactan from *B. occidentalis* inhibits intrinsic tenase and prothrombinase complexes that are critical for factor Xa and thrombin generation, respectively [130]. The sulfated galactan interacts with the heparin-binding site on the heavy chain of factor Xa. Interestingly, the anticoagulant activities associated with the sulfated galactan and that of heparin are modulated differently by heparin cofactor II; heparin anticoagulant activity was enhanced in plasma devoid of heparin cofactor II, whereas the activity of the sulfated galactan was independent of this cofactor. 

Heparin is used extensively for the prevention of venous thrombosis and the treatment of other thromboembolic disorders due to its inhibition of thrombin and other enzymes in the coagulation system. To overcome the obvious potential side-effect of bleeding, researchers have investigated means of reducing the anticoagulant activities of heparin while enhancing its anti-thrombotic activities including chemical modification and fractionation of native heparin to lower molecular forms [132,133,134]. Nevertheless, the development of antithrombotic algal polysaccharides would be advantageous since their use would avoid the potential for contamination with prions or viruses in commercial heparins, which are obtained from pig and bovine intestine. Moreover, with more specific activities and/or targets, the algal sulfated polysaccharides could find applications complementary to heparin [132]. To this end, one of the approaches has been to develop low molecular weight (LMW) fucoidans [121,135]. For example, a LMW fraction of approximately 8,000 derived from the fucoidan of *A. nodosum* reduced mean thrombus weight by 80%*vs.* control saline injection in a rabbit model of venous thrombosis [135]. This LMW fucoidan and related derivatives [136] are promising since they show lower effects in coagulation tests when compared to the commercial LMW heparin, dalterparin (Fragmen®, Pfizer Inc.). More recently, Rocha and colleagues reported that a sulfated galactofucan from the brown seaweed *Spatoglossum schoederi* has potent antithrombotic activity in a rat model of venous thrombosis [69]. Unlike heparin, which produces a rapid but transient antithrombotic effect, the *in vivo* action of this sulfated galactofucan progressed slowly, showing maximal effectiveness about eight hours post injection. When tested *in vitro* using endothelial cells, it was discovered that the galactofucan stimulates the production of heparan sulfate leading to the hypothesis that its delayed action *in vivo* is tied to the need for an accumulation of the heparan sulfate on blood vessel surfaces. Despite its high sulfation level, the galactofucan lacks significant anticoagulation activity, making it an ideal candidate as an antithrombotic agent [69]. 

### 4.2. Antiviral Activity

The ability of sulfated polysaccharides from seaweeds to inhibit the replication of enveloped viruses including herpes simplex virus (HSV), human immunodeficiency virus (HIV), human cytomegalovirus, dengue virus and respiratory syncytial virus is well established [137,138,139,140]. The original observations on antiviral activities of seaweed constituents go back more than 50 years to the observation that seaweed extracts protected chicken embryos against influenza B and mumps [141]. 

It was discovered a little later, somewhat serendipitously, that heparin inhibited HSV in leukocyte cultures [142], an effect hypothesized to be due to electrostatic interference with viral attachment to the cell surfaces. This spurred research into the antiviral effects of various polyanionic substances including sulfated polysaccharides from a number of seaweed species. Interest around the antiviral effects of algal polysaccharides has grown substantially in recent years with the mounting evidence that their effects on viral replication occurs by a number of mechanisms that involve specific structural qualities of the polysaccharides and not simply through non-specific interactions [137,143]. We will discuss some of the more recent findings regarding the structural requirements of algal polysaccharides for antiviral activity and the underlying modes of action. 

A fucan from *Cladosiphon okamuranus* composed of glucuronic acid and sulfated fucose units potently inhibited infection of BHK-21 cells with dengue virus type 2 (DENV-2), but showed little effect on the other three serotypes of the virus [144]. Sulfation of the fucan was necessary for this activity; surprisingly, carboxyl-reduction of the glucuronic acids to glucose units also abolished the fucans’ antiviral properties. Analysis of the structure of the envelope glycoproteins from the four serotypes of dengue virus suggested that arginine-323 in DENV-2, which is proximal to the putative heparin binding site, was critical for the interaction with the fucan. 

Similarly, Talarico and colleagues reported that two sulfated polysaccharides from red seaweeds, a carrageenan from *Gymnogongrus griffithsiae* and a galactan from *Cryptonemia crenulata*, inhibited DENV-2 multiplication in Vero cells [145,146,147]. Their effects were less potent against DENV-3 and DENV-4 and were completely inactive against DENV-1 infection [147]. These polysaccharides were shown to interfere with both DENV-2 adsorption and internalization into the cells and were only effective if added together with the virus or shortly after infection. For example, no inhibition of virus multiplication occurred when the normal viral entry process was bypassed by DENV-2 RNA transfection into the cells [145,146].

Recently, light has also been shed on the antiviral activity of marine sulfated polysaccharides against HSV types 1 and 2 (HSV-1, HSV-2) [67,143,148,149]. Sulfated xylomannans from the red seaweed *Sebdenia polydactyla* inhibited the propagation of HSV-1 in Vero cells [148]. The activity was abolished by desulfation of the xylomannan and, conversely, oversulfated derivatives exhibited enhanced potency. Mohsen reported that sulfated polysaccharide fractions isolated from *Sargassum latifolium* inhibited HSV-1 in the plaque assay with the most effective fraction having greater sulfate ester content and molecular weight compared to the other fractions studied [150]. It has been generally observed that antiviral activity of sulfated polysaccharides increases with their molecular weight [137]. Representative polysaccharides from brown and red seaweeds differing in structure (galactans, fucans and galactofucans), sulfation level and molecular weight were shown to inhibit HSV-1 and HSV-2 infection [149] reinforcing the view that, as with other activities, antiviral activity of sulfated polysaccharides is due to a complex interplay of structural features including sulfation level, distribution of sulfate groups along the polysaccharide backbone, molecular weight, sugar residue composition, and stereochemistry [139,143]. A subset of these sulfated polysaccharides also showed an ability to inactivate HSV-2 directly through incubation with the virus. This virucidal activity has significance since it is associated with augmentation of antiviral activity *in vivo* [151].

### 4.3. Immuno-Inflammatory Activity

Sulfated polysaccharides, including those from algae, have been shown to possess immunomodulatory activities that may be of potential application in stimulating the immune response or in controlling immune cell activity to mitigate associated negative effects such as inflammation [152]. Sulfated polysaccharides may affect multiple targets in the immune and inflammatory systems that can have impact on disease progression and outcome including tumor progression and metastasis [153]. 

One of the interests in algal sulfated polysaccharides as anti-inflammatory agents is the growing body of evidence illustrating their ability to interfere with the migration of leukocytes to sites of inflammation. For example, in a rabbit model of bacterial meningitis, leukocyte rolling was markedly reduced by intravenous infusion of fucoidan [154]. Similarly, intravenous addition of fucoidan reduced, in a dose-dependent manner, leukocyte recruitment to peritoneum in a rat model of peritoneal inflammation [155]. These effects were ascribed to the binding of fucoidan to L- and P selectins, cell adhesion molecules essential in the recruitment process. Both of these studies used the fucoidan from Sigma-Aldrich Chemical Co (St. Louis, MO, U.S.) that is sourced from *F. vesiculosus*. Fucans from other seaweeds including *Laminaria sp* *p*., *Fucus sp* *p*., *A. nodosum*, and *C. okamuranus* also inhibit leukocyte recruitment to the abdominal cavity during acute peritonitis in rats [122]. In addition to impairing the action of selectins, algal sulfated polysaccharides inhibit tissue degradative enzymes such as heparanase and elastases that are involved in the breakdown of basement membrane integrity during inflammation [156,157]. 

One of the major and potentially promising activities is the potent inhibitory effect of sulfated fucans on human complement activation. The original observations showed that fucoidan fractions from *A. nodosum* potently inhibit both the classical and alternative pathways in human serum [158]. Tissot and colleagues have extensively studied this activity [159,160,161,162]. It was determined that low molecular weight fucoidan fractions bind to the C1q subunit of the C1 complex that triggers complement through recognition and binding of immune complexes [162]. The binding of fucoidan appears to interfere with the ability of C1q to fully trigger C1 activation [160]. Fucoidan also binds C4, thereby preventing its breakdown and generation of its cleavage product C4b, the latter being required for the formation of C3 convertase and the propagation of complement [162]. Furthermore, it was found that fucoidan binds C1q globular heads and may interfere with C1q recognition of IgG [161]. Recently, using NMR, it was found that branched fucoidan oligosaccharides are better at inhibiting complement compared to linear structures [159]. 

The interaction of algal sulfated polysaccharides with the complement system suggests that they may have utility in influencing innate immunity to reduce the pro-inflammatory state or other detrimental conditions such as allergic reactions arising during the innate immune response. In addition, there is a growing body of evidence that algal polysaccharides can regulate the innate immune response directly by binding to pattern recognition receptors (PRRs) such as the mannose receptor and toll-like receptors on phagocytic cells including macrophages [152]. For example, λ-carrageenan stimulated mouse T cell cultures in a toll-like receptor-4 (TLR4) dependent manner [163] generating a T helper 1 (Th1) patterned cytokine response. However, splenocytes prepared from TLR4-deficient mice still retained some ability to produce interferon-γ in response to λ-carrageenan suggesting that PRRs other than TLR4 were also elicited. In mice immunized with ovalbumin to produce an allergic reaction, oral dosing with λ-carrageenan lead to a reduction in ovalbumin-specific IgE and serum histamine release, suggesting that λ-carrageenan might be used to ameliorate allergic reactions. Similar results were reported for mekabu fucoidan from *U. pinnatifida* [164]. 

Direct stimulatory effects of algal polysaccharides on immune cells results in production of nitric oxide through induction of inducible nitric oxide synthase (iNOS) and a pro-inflammatory cytokine/chemokine profile [165]. Depending on the situation, the interaction of sulfated polysaccharides with other effectors may result in reduced inflammation. For example, fucoidan from *F. vesiculosus* induced iNOS in RAW264.7 macrophage cells leading to enhanced production of nitric oxide [166,167]. Yet, in the presence of lipopolysaccharide (LPS), the fucoidan impaired LPS-induced expression of iNOS and nitric oxide production [167]. Similarly, fucoidan suppresses interferon gamma-induced iNOS expression in macrophage and glial cell types [168]. 

These and other reports of algal sulfated polysaccharides directly stimulating the innate immune system [165,168,169,170] suggests that they may find therapeutic use in opposing T helper 2 (Th2)-based pathologies such as autoimmune disorders and allergy. Additionally, there is evidence that algal sulfated polysaccharides including fucoidans and carrageenans increase the cytotoxicity of natural killer cells, lymphocytes and macrophages against tumors [169,171]. 

The structural requirements for this immunostimulatory activity of algal sulfated polysaccharides have not been greatly studied. One report, by Leiro and colleagues, has shown greatly diminished immunostimulatory activity of ulvan-like polysaccharides from *U. rigida* when they were desulfated [165]. 

### 4.4. Antioxidant Activities

Algal sulfated polysaccharides, until recently, were largely ignored as sources of antioxidant activity. Studies over the last several years reveal that sulfated polysaccharides from a number of seaweeds have appreciable antioxidant capability [95,172,173,174,175,176,177]. For example, fucans from *F. vesiculosus* exhibited considerable ferric reducing/antioxidant power [172] and superoxide radical scavenging ability [173]. Fucan fractions from *L. japonica* also showed significant antioxidant capabilities in superoxide radical and hydroxyl radical scavenging assays [174,176,177]. Superoxide radical scavenging activity correlated positively with the sulfate content of the polysaccharide fractions [173,176]. Antioxidant properties of carrageenans [173] and ulvans [94] also appeared related to sulfate content. In the latter study, high sulfate content derivatives of ulvan showed improved antioxidant activities [94]. Interestingly, metal chelating, free radical and hydroxyl radical scavenging activities of fucan fractions appear to relate to their ratio of sulfate content/fucose [176]. 

### 4.5. Antilipidemic Effects

Algal sulfated polysaccharides exert lipid-lowering and other beneficial properties in hyperlipidemic animal models [178,179,180]. An extract from *F. vesiculosus*, in a dose-dependent manner, effectively reduced the elevation in serum triglyceride and total cholesterol levels in triton-induced-hyperlipemic rats. In rats fed a high cholesterol diet for 21 days, supplementation of the diet with ulvan from *U. pertusa* led to reductions in serum total cholesterol and LDL-cholesterol with no significant alteration in serum triglycerides [180]. The effects of ulvan were modified when it was degraded into lower molecular weight fractions. Ulvan derivatives of lower molecular weight and intrinsic viscosity did not reduce serum cholesterol but did normalize the hypertriglyceridemia of these animals and raised HDL-cholesterol. The underlying mechanisms of these actions are unclear but it does not appear to involve bile acid sequestration since ulvan and its lower molecular weight derivatives increased bile excretion to a similar extent. 

Recently, it was reported that fucoidan from *L. japonica* reduced serum total and LDL-cholesterol and triglycerides and raised HDL-cholesterol in a hyperlipidemic rat model [179]. The treatment also increased the activities of lipoprotein lipase (LPL), hepatic lipase (HL) and lecithin cholesterol acyltranferase (LCAT) in serum. These changes in enzyme activities could be the direct result of fucoidan treatment or an indirect effect associated with improvement in lipid profile. Certainly fucoidan and other algal sulfated polysaccharides may influence LPL and HL through interaction with well-characterized heparin-binding sites on these enzymes. Consistent with this is the observation that fucoidan from *F. vesiculosus* releases LPL from cell surface binding sites and stabilizes LPL activity in culture medium [181]. 

Algal sulfated polysaccharides are showing promising effects in addressing the hyperlipidemia associated with certain drug toxicities. Fucans from *S. polycystum* were shown to have significant preventive effects on the elevation of cholesterol and triglycerides in serum and liver tissue resulting from acetaminophen-induced toxic hepatitis [182]. Treatment also partially reversed the reduction in hepatic LCAT and HL and improved overall histological appearance of the liver. Similarly, a sulfated polysaccharide from *S. wightii* reduced hyperlipidemia and normalized LPL and LCAT in plasma in cyclosporine A-induced nephrotoxicity [183]. The excretion of urea, uric acid, and creatinine were normalized by the sulfated polysaccharide treatment. In addition, the susceptibility of LDL to oxidation was reduced, suggesting that the antioxidant activity of the sulfated polysaccharide was also playing a role and may contribute to its renoprotective activity. 

## 5. Future Perspectives

Algal sulfated polysaccharides are a source of numerous biological activities that may find therapeutic benefit. They are structurally diverse and heterogeneous, which makes studies of their structures challenging, and may also have hindered their development as therapeutic agents to date. The production of a standardized commercial product based on algal sulfated polysaccharide constituents will be a challenge since their structural and pharmacological features may vary depending on species and on location and time of harvest. For example, Bourgougnon and colleagues reported that there was a significant annual variation in the composition and the *in vitro* anti-HIV-1 activity of a water-soluble sulfated glucuronogalactan from *Schizymenia dubyi* [184]. Another issue to the therapeutic use of algal polysaccharides is their potentially low bioavailability given their often high molecular weights. It is likely, based on observations with heparin [185], that algal sulfated polysaccharides will display some, albeit low, degree of oral bioavailability. A recent pilot study in humans reported that fucoidan was ineffective as an oral anticoagulant agent [186], which underscores the issue. It also emphasizes the importance of understanding the structural requirements for biological activity and whether low molecular weight derivatives, which are potentially more bioavailable, remain active. For some applications, low bioavailability may not be a concern. First, some of the hypolipidemic effects of seaweed sulfated polysaccharides arise through effects on bile acid sequestration in the intestinal lumen. Second, for some immunomodulatory activities, the site of activation of the immune system may also be within the intestinal lumen (e.g., at Peyer’s patches) as has been hypothesized for immunomodulatory effects of polysaccharide constituents from *Chlorella pyrenoidosa* [187]. Finally, algal sulfated polysaccharides are already used topically in cosmetics and there is significant interest in further development for cosmetics and cosmeceuticals products [188]. 
